# Assessment of Optical and Phonon Characteristics in MOCVD-Grown (Al_x_Ga_1−x_)_0.5_In_0.5_P/n^+^-GaAs Epifilms

**DOI:** 10.3390/molecules29174188

**Published:** 2024-09-04

**Authors:** Devki N. Talwar, Zhe Chuan Feng

**Affiliations:** 1 Department of Physics, University of North Florida, 1 UNF Drive, Jacksonville, FL 32224-7699, USA; 2 Department of Physics, Indiana University of Pennsylvania, 975 Oakland Avenue, 56 Weyandt Hall, Indiana, PA 15705-1087, USA; 3 Southern Polytechnic College of Engineering and Engineering Technology, Kennesaw State University, Marietta, GA 30060, USA; zfeng6@kennesaw.edu

**Keywords:** (Al_x_Ga_1−x_)_y_In_1−y_P alloys, MOCVD-grown epilayers, photoluminescence, Raman scattering, Fourier-transform infrared spectroscopy, macroscopic models

## Abstract

Quaternary (Al_x_Ga_1−x_)_y_In_1−y_P alloys grown on GaAs substrates have recently gained considerable interest in photonics for improving visible light-emitting diodes, laser diodes, and photodetectors. With two degrees of freedom (x, y) and keeping growth on a lattice-matched GaAs substrate, the (Al_x_Ga_1−x_)_0.5_In_0.5_P alloys are used for tuning structural, phonon, and optical characteristics in different energy regions from far-infrared (FIR) → near-infrared (NIR) → ultraviolet (UV). Despite the successful growth of (Al_x_Ga_1−x_)_0.5_In_0.5_P/n^+^-GaAs epilayers, limited optical, phonon, and structural characteristics exist. Here, we report our results of carefully examined optical and vibrational properties on highly disordered alloys using temperature-dependent photoluminescence (TD-PL), Raman scattering spectroscopy (RSS), and Fourier-transform infrared reflectivity (FTIR). Macroscopic models were meticulously employed to analyze the TD-PL, RSS, and FTIR data of the (Al_0.24_Ga_0.76_)_0.5_In_0.5_P/n^+^-GaAs epilayers to comprehend the energy-dependent characteristics. The Raman scattering and FTIR results of phonons helped analyze the reflectivity spectra in the FIR region. Optical constants were carefully integrated in the transfer matrix method for evaluating the reflectivity R(E) and transmission T(E) spectra in the NIR → UV regions, validating the TD-PL measurements of bandgap energies $\left(E_{g}^{PL}\right)$.

## 1. Introduction

Epitaxially grown quaternary (Al_x_Ga_1−x_)_y_In_1−y_P/n^+^-GaAs epilayers with many flexible parameters are considered technologically attractive material systems [1,2,3,4,5,6,7,8,9,10,11]. The alloys of (Al_x_Ga_1−x_)_y_In_1−y_P (referred to as AlGaInP) are frequently described as the combination of AlInP and GaInP, offering many options to crystal growers for designing various electronic devices using low-dimensional heterostructures (LDHs) by adjusting dopants, compositions x, y, and film thickness d. With a fixed value of y = 0.51 and varying x, the quaternary alloys can attain a range of direct energy bandgaps $E_{g}$ (≡1.91−2.37 eV) and diverse values of dielectric functions $\tilde{\varepsilon }\left(E\right)$ and/or refractive indices ñ(E) [12,13,14,15,16,17,18,19,20,21,22,23,24,25,26,27,28,29,30,31]. Subsequent modifications in lattice constants $a_{o}$ allowed an effective growth of AlGaInP alloys on lattice-matched GaAs and/or Ge substrates [12,13,14]. For many years, efforts have been made to prepare different III-V alloys by intermixing two or more binary compounds. For instance, ternary alloys (In_x_Ga_1−x_P, Al_x_Ga_1−x_P or InAs_x_P_1−x_, GaAs_x_P_1−x_) are acquired by combining two binary materials having either common anions or common cations. Quaternary alloys (Al_x_Ga_y_In_1−x−y_P, Al_x_Ga_y_In_1−x−y_As or Al_x_Ga_1−x_As_y_P_1−y_, In_x_Ga_1−x_As_y_P_1−y_) are also created by incorporating three or four binary compounds. These possibilities have motivated many scientists and engineers to consider different epitaxial methods (viz., molecular beam epitaxy (MBE) [17,18,32,33], metal-organic vapor phase epitaxy (MOVPE), and metal-organic chemical vapor deposition (MOCVD)) for preparing ultrathin (Al_x_Ga_1−x_)_y_In_1−y_P/n^+^-GaAs epilayers, multi-quantum wells (MQWs), and superlattices (SLs) [34,35,36].

AlGaInP-based LDHs have been recognized as exceptionally promising materials for designing and/or developing high-electron-mobility transistors (HEMTs), heterostructure-based bipolar transistors (HBTs), laser diodes (LDs), photodetectors (PDs), visible light-emitting diodes (LEDs), frequency-mixing components (FMCs), electro-optic modulators (EOMs), multi-junction solar cells (MJ-SCs), solid-state emitters (SSEs), integrated circuits (ICs) [1,2,3,4,5,6,7,8,9,10,11,12,13,14,15,16,17,18,19,20,21,22,23,24,25,26,27,28,29,30,31], etc. While laser emission in the red spectral region was demonstrated recently from (Al_x_Ga_1−x_)_y_In_1−y_P alloys with y ~ 0.5, highly efficient LEDs, emitting green-colored light in the visible spectral range, have already been manufactured and are available commercially [37,38,39,40,41,42,43,44,45,46,47,48,49,50]. Now, these devices are being incorporated as inter-connects or sensors in different nano-/micro-electronics as well as in photonic systems to meet the growing strategic and civilian application needs for space exploration, energy, and the bio-medical arena for diagnoses, drug analyses/treatments, etc. Despite many efforts made in engineering electronic devices comprising (Al_x_Ga_1−x_)_y_In_1−y_P/GaAs epifilms, MQWs, and/or SLs, the optical, thermodynamic, structural, and phonon traits of the LDHSs are, however, still not adequately appraised using experimental and/or theoretical methods in the far-infrared (FIR) → near-infrared (NIR) → ultraviolet (UV) energy regions [51,52,53,54,55,56,57,58,59,60,61,62,63,64,65,66,67,68,69,70,71,72,73].

Research on the optical and phonon properties of semiconductor materials (viz., absorption coefficient α(E), reflectance R(E), transmittance T(E), etc.) has gained significant interest owing to their importance in optoelectronics, photonics, and quantum technologies. These properties can be achieved by careful evaluations of the complex dielectric functions $\tilde{\varepsilon }(\lambda \;or\;E)$ [7,8,9,10,11,12,13]. Specifically, α(E) in a material represents the penetration depth of photons at any wavelength before they are absorbed and/or transmitted. The accurate selection of material can impact the performance of electronic devices. This is simply because α(E) predicts the minimum detectivity of a material and plays a crucial role in designing high-performance lasers, sensors, imaging devices, information processing, communication tools, and energy harvesters [1,2,3,4,5,6,7,8,9,10,11,12,13,14,15,16,17,18,19,20,21,22,23,24,25,26,27,28,29,30,31]. Many semiconductors have shown the ability to detect different groups of light depending upon their bandgap energies $E_{g}$. The evaluations of $\tilde{\varepsilon }\left(E\right),$ for binary compounds and alloys in different wavelengths, are closely related to their phonon frequencies and inter-band transition energies at high critical points in the Brillouin zone (BZ). The optical response of a material can be fully accomplished by using $\tilde{\varepsilon }\left(E\right)[\equiv \varepsilon_{1}\left(E\right)+i\varepsilon_{2}\left(E\right)$] and relating its real [$\varepsilon_{1}\left(E\right)$] and imaginary [$\varepsilon_{2}\left(E\right)$] parts to the Kramers–Krönig transformation. As the structural, phonon, and electrical attributes of LDHs are considered vital in device designs, it is therefore a customary tradition to probe these traits by exploiting a variety of characterization techniques [51,52,53,54,55,56,57,58,59,60,61,62,63].

Many experimental methods [37,38,39,40,41,42,43,44,45,46,47,48,49,50,51,52,53,54,55,56,57,58,59,60,61,62,63,64], including high-resolution X-ray diffraction (HR-XRD), energy-dispersive X-ray (EDX) [46], reflectance anisotropy (RA) [47], Auger spectroscopy, secondary ion mass spectroscopy (SIMS), Fourier-transform infrared (FTIR) [38,39] spectroscopy, Raman scattering spectroscopy (RSS) [37,42], photoluminescence (PL) [37,40,51,52,53,54,55], atomic force microscopy (AFM), photo reflectance (PR), reflection high-energy electron diffraction (RHEED) [57,58,59], scanning electron microscopy (SEM), inelastic neutron scattering (INS) [60,61,62], and spectroscopic ellipsometry (SE) [44,56], are employed for assessing LDH phonon, electrical, and structural characteristics. While the HR-XRD, FTIR [38,39], and RSS methods have played [37,40,51,52,53,54,55] important roles in appraising the structural, phonon, and optical characteristics in bulk materials, their exploitations to assess film thickness d, interfacial structure, and surface relaxation in LDHs (e.g., MQWs and SLs) have remained surprisingly enigmatic. Despite the limited SE reports [29,44,56] on the (Al_x_Ga_1−x_)_y_In_1−y_P/GaAs system, no systematic experimental and/or theoretical efforts have been made for comprehending their phonon, structural, and optical characteristics.

This paper aims to report the results of methodical experimental and theoretical efforts that we have formulated for carefully analyzing PL, RSS, and FTIR data to evaluate the optical, phonon, and structural characteristics (cf. Section 3, Section 3.1, Section 3.2, Section 3.3 and Section 3.4) of (Al_x_Ga_1−x_)_y_In_1−y_P/n^+^-GaAs epilayers. A set of four (Al_x_Ga_1−x_)_y_In_1−y_P samples, T_i_ (i = 1–4; x = 0.24, y = 0.5) (see Table 1), of different thicknesses d (≡0.85 μm–1.08 μm) were prepared using an MOCVD method (cf. Section 3.1) in the vertical growth setting. Temperature-dependent PL (TD-PL) measurements were performed (cf. Section 3.2) on each sample by using a 532 nm excitation source. The luminescence dispersed by a monochromator detector was carefully collected using a liquid-nitrogen-cooled InSb detector. Raman scattering spectroscopy measurements (cf. Section 3.3) on these samples were accomplished in the backscattering configuration by exploiting a Renishaw spectrometer and using a He-Ne laser source with 633 nm as the excitation. Fourier-transform infrared reflectivity studies in the FIR region were achieved (cf. Section 3.4) at near-normal incidence (θ = 0) by using a high-resolution Brüker IFS 120 v/S spectrometer for assessing the composition- and thickness-dependent variation in reflectivity $R\left(\omega \right)$ spectra on (cf. Section 3.4) the MOCVD-grown epifilms. Appropriate macroscopic models were adopted for carefully analyzing the experimental (PL, Raman scattering, and FTIR) data for appraising energy-dependent optical responses on the (Al_x_Ga_1−x_)_0.5_In_0.5_P/n^+^-GaAs epilayers. Systematic results of numerical simulations based on different macroscopic methods [64] were achieved and are compared/contrasted against the experimental/theoretical data (c.f. Section 2, Section 2.1, Section 2.2, Section 2.3, Section 2.4 and Section 2.5). In the NIR → UV energy regions, we used an MDF formalism for accurately calculating the refractive indices n(E) and extinction coefficients κ(E). These optical constants were incorporated in the transfer matrix method (TMM) to calculate the reflectivity $R\left(E\right)$ and transmission T(E) spectra of ultrathin (Al_0.24_Ga_0.76_)_0.5_In_0.5_P/n^+^-GaAs samples (cf. Section 2.5.1, Section 2.5.2, Section 2.5.3 and Section 2.5.4). The estimated energy bandgaps from $R\left(E\right)$/T(E) of (Al_0.24_Ga_0.76_)_0.5_In_0.5_P/n^+^-GaAs epilayers agreed reasonably well with the PL data. The conclusions drawn from this study are summarized in Section 4. The optical, structural, and phonon characteristics using different analytical techniques on the (Al_x_Ga_1−x_)_0.5_In_0.5_P/n^+^-GaAs epilayers provided good information on the quality of the samples. These methods can be extended to evaluate other low-dimensional heterostructure materials of technological interest.

## 2. Results and Discussions

Customarily, III-V compound semiconductor-based quaternary (Al_x_Ga_1−x_)_0.5_In_0.5_P/n^+^-GaAs alloy epilayers of different composition, dopant types, thicknesses, and order parameters are used in many optoelectronic and photovoltaic devices [6,7]. Long-range chemical collation is normally anticipated. A CuPt-type ordering may occur in these materials due to surface reconstruction during their growth. This ordering can induce a lowering of symmetry, which can cause small birefringence in the dielectric function of epilayers in the near-bandgap spectral region [29]. Such an elusive analysis is beyond the scope of this study. Hence, the anisotropy is not considered here for data treatment in simulating phonon/optical characteristics. The relevance of isotropic approximation in III–V quaternary epilayers has already been reported by many researchers in different published works [29].

### 2.1. Energy Bandgap by Photoluminescence

**Figure 1:**
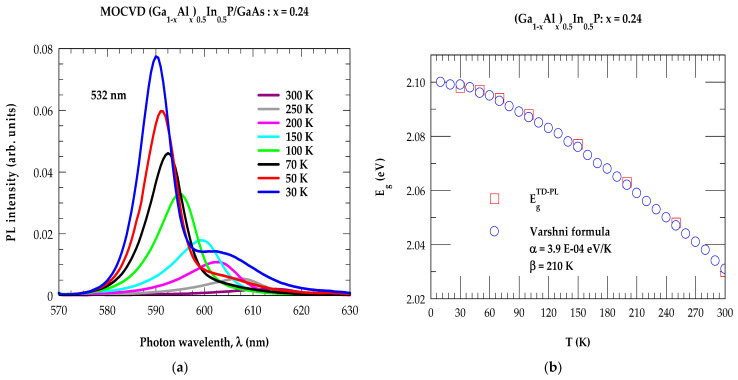
(**a**) Temperature-dependent photoluminescence measurements exhibiting a shift in intensity peaks of electronic energy bandgaps ($E_{g}^{PL}$) from 2.1 eV to 2.03 eV, in good agreement with the experimental/theoretical results. (**b**) Comparison of the experimental (open red color square) energy bandgap of the (Al_0.24_Ga_0.76_)_0.50_In_0.50_P alloy with Varshni’s (Ref. [70]) formula (blue open circles) using an appropriate set of values for the adjustable parameters α and β (see text).

By using a HORIBA Jobin Yvon T64000 micro-Raman system, we report in Figure 1a the PL results acquired in the spectral range λ (≡570 nm–630 nm) exploiting a 532 nm excitation source. As the temperature, T (≡30 K–300 K), changes, the PL intensity measurements on the (Al_0.24_Ga_0.76_)_0.50_ In_0.50_P/n^+^-GaAs epilayer sample #T_2_ revealed appropriate shifts with the decrease in peak intensities exhibiting electronic energy bandgaps $E_{g}^{PL}$ (≡2.1 eV to 2.03 eV), in excellent agreement with the existing experimental/theoretical [29] data as well as the results derived by using the Varshni formula [70] (cf. Section 2.1.1). The decrease in PL intensities with the increase in T in our samples is due to the enhancement of non-radiative recombination processes.

#### 2.1.1. Temperature-Dependent Energy Bandgap

In a semiconductor alloy, the temperature-dependent energy bandgap can be estimated by using the Varshni formula [70]:

(1) $$E_{g}\left(T\right)=E_{g}\left(T\to 0\right)-\frac{\alpha T^{2}}{T+\beta },$$

where α and β are the fitting parameters characteristic of a given material. With the choice of α = 3.9 E-4 eV/K and β = 210 K for the quaternary (Al_0.24_Ga_0.76_)_0.50_ In_0.50_P alloy, our calculated T-dependent energy bandgaps (see Figure 1b) compared favorably well with the experimental TD-PL data.

#### 2.1.2. Composition-Dependent Energy Bandgap

**Figure 2:**
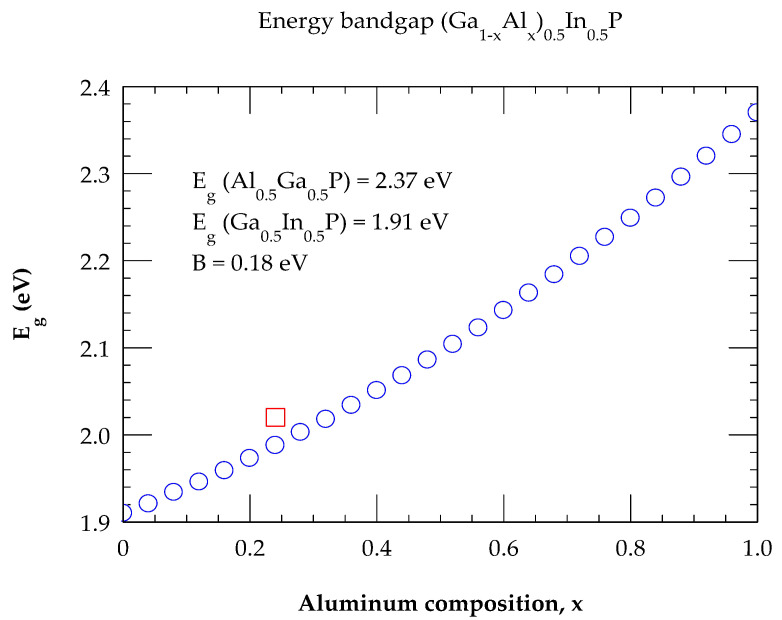
Composition-dependent energy bandgap of (Al_x_Ga_1−x_)_0.5_In_0.5_P alloys as a function of composition by using Equation (2) following Ref. [71]. The PL result of E_g_ at x = 0.24 is shown by red color open square.

The composition-dependent energy bandgaps of quaternary (Al_x_Ga_1−x_)_0.5_In_0.5_P alloys can be evaluated by exploiting the $E_{g}$ values of ternary ${Al}_{0.5}{Ga}_{0.5}P$ and ${Ga}_{0.5}{In}_{0.5}P$ alloys using the following equation [71]:

(2) $$E_{g}{\left({Al}_{x}{Ga}_{1-x}\right)}_{0.5}{In}_{0.5}P=xE_{g}\left({Al}_{0.5}{Ga}_{0.5}P\right)+\left(1-x\right)E_{g}\left({Ga}_{0.5}{In}_{0.5}P\right)-x\left(1-x\right)B,$$

where B is the bowing parameter. With the appropriate selection of $E_{g}$ values for ${Al}_{0.5}{Ga}_{0.5}P,\;{Ga}_{0.5}{In}_{0.5}P$ alloys and B (≡0.18 eV), the calculated results of the bandgap for ${\left({Al}_{x}{Ga}_{1-x}\right)}_{0.5}{In}_{0.5}P$ displayed in Figure 2 agreed quite well with the experimental data for x~0.24.

Again, the TD-PL results will be analyzed in Section 2.5, Section 2.5.1, Section 2.5.2, Section 2.5.3 and Section 2.5.4 by using a comprehensive MDF formalism [29] and simulating the optical constants (i.e., refractive indices n(E), extinction coefficients $\kappa \left(E\right)$) in the NIR → UV energy regions for the (Al_0.24_Ga_0.76_)_0.5_In_0.5_P alloy and GaAs. Incorporating n(E) and $\kappa \left(E\right)$ for the alloy and substrate along with film thickness d, our simulated $R(E)/T\left(E\right)$ spectra using the TMM approach offered the energy bandgap $E_{g}^{PL}$ for a 0.9 μm thick epilayer, in excellent agreement with the PL measurements.

### 2.2. Optical Phonons by Raman Scattering Spectroscopy

**Figure 3:**
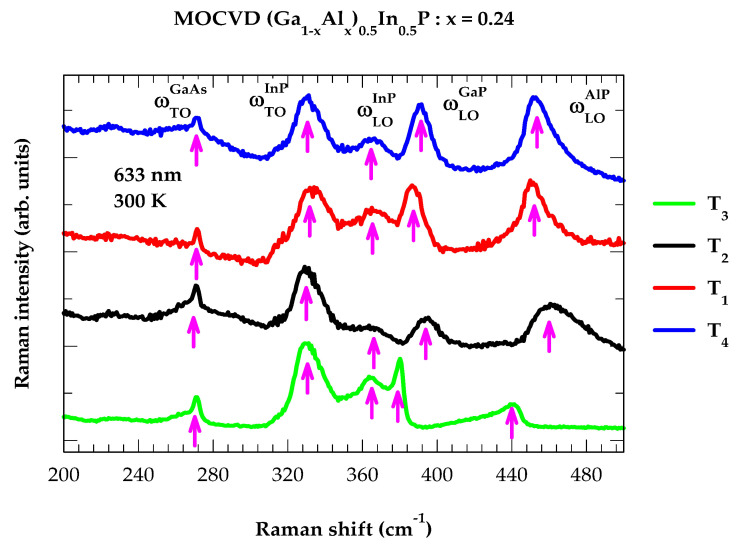
Raman scattering spectra of four MOCVD-grown (Al_x_Ga_1−x_)_0.5_In_0.5_P/n^+^-GaAs epilayers with x = 0.24. These measurements were performed by using a Renishaw Raman Microscope model-100 in the backscattering geometry with a 633 nm laser beam from a He–Ne source. Vertical magenta-colored arrows are used to indicate the TO mode of the GaAs (substrate) as well as the AlP-, GaP-, and InP-like LO and TO modes of the quaternary alloy (see text).

Many systematic analyses of the RSS spectra have been reported in different semiconductor epilayers for comprehending their structural (i.e., strain due to lattice mismatch), chemical (composition), and configurational (ordering) characteristics [37,42]. In Figure 3, we have reported our Raman scattering measurements on four MOCVD-grown (Al_x_Ga_1−x_)_0.5_In_0.5_P/n^+^-GaAs epilayers of composition x = ~0.24. Vertical magenta-colored arrows are drawn in Figure 3 to indicate the values of major optical phonon energy features for both the epifilms and substrate (see Table 2). Like PL experiments, one expects the Raman results of the (Al_x_Ga_1−x_)_0.5_In_0.5_P alloys to be dependent on the RSS data of the ternary In_y_Al_l−y_P and In_y_Ga_l−y_P alloys.

In addition to observing the $\omega_{TO}$ mode (≡~269 cm^−1^) of the GaAs substrate, the Raman measurements identified four additional phonon features. The frequencies include two InP-like ($\omega_{TO}$, $\omega_{LO}$), one GaP-like ($\omega_{LO}$), and one AlP-like ($\omega_{LO}$) mode, respectively. Slight variations in the frequencies reflect disorder-related effects. Raman scattering studies performed earlier on MBE-grown In_0.48_Ga_0.52_P/GaAs epilayers [49,51,52,53,54] perceived three vibrational features: two $\omega_{LO}$ (GaP-like, InP-like) modes at ~380 cm^−1^, ~360 cm^−1^ and one $\omega_{TO}$ (InP-like) mode at ~330 cm^−1^. Similar studies on MOCVD-grown In_0.5_Al_0.5_P/n^+^-GaAs epitaxial layers [55] revealed two optical phonon features (InP-like) at ~340 cm^−1^ and (AlP-like) ~460 cm^−1^, respectively. A comparison with our measurements on quaternary alloys (see Table 2) clearly corroborated the dependence of RSS results on the ternary In_y_Al_l−y_P and In_y_Ga_l−y_P alloys.

### 2.3. Optical Phonons by Far-Infrared Spectroscopy

**Figure 4:**
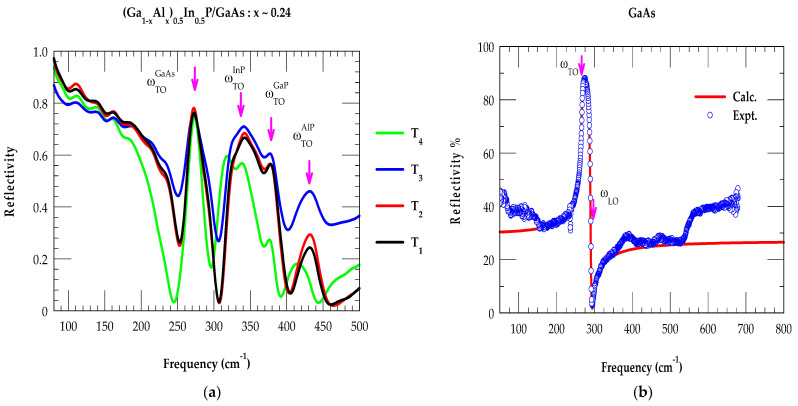
(**a**) Experimental reflectivity spectra using a high-resolution Brüker IFS 120 v/S FTIR spectrometer on four MOCVD-grown (Ga_1−x_Al_x_)_0.5_In_0.5_P/GaAs T_i_ (i = 1, 4) samples with x = 0.24 (see Table 1). Magenta vertical arrows identify the major optical modes. (**b**) Experimental (calculated) reflectivity spectra of n^+^ GaAs substrate using blue-colored open circles (red-colored line) (see text).

Room-temperature FTIR measurements were performed both on (Al_0.24_Ga_0.76_)_0.5_In_0.5_P/n^+^-GaAs epifilms (cf. Section 3.1) and the GaAs substrate by using a high-resolution Brüker IFS 120 v/S FTIR spectrometer. The results are reported in Figure 4a,b for the four T_i_ (i = 1, 4) samples and the substrate, respectively. During the growth of MOCVD epilayers of thickness d (between 0.85 μm and 1.1 μm), the samples were doped (see Table 1) either by incorporating Si or Te. In the binary zb (GaAs) material, one expects a TO and an LO mode (magenta-colored vertical arrows in Figure 4b). The $\omega_{TO},$ $\omega_{LO}$ frequencies are related to the Lyddane–Sachs–Teller relation $\left(\frac{\varepsilon_{0}}{\varepsilon_{\infty }}=\frac{\omega_{LO}^{2}}{\omega_{TO}^{2}}\right)$, where the terms $\varepsilon_{0}$ and $\varepsilon_{\infty }$ are the static and high-frequency dielectric constants, respectively.

The reflectivity spectrum of GaAs (Figure 4b) is seen dropping to a minimum at the plasma edge (${near\;\omega }_{LO}$ ~293 cm^−1^). Its position depends on the carrier concentration while revealing a sharp peak near the $\omega_{TO}$ (~269 cm^−1^) mode. In the Al_0.24_Ga_0.76_)_0.5_In_0.5_P/n^+^-GaAs epilayers, the $\omega_{TO}$ phonon frequencies (see Figure 4a) of the GaAs-like, InP-like, GaP-like, and AlP-like modes (see Table 3) are shown by using magenta-colored vertical arrows. Like Raman scattering, the FTIR reflectivity results of (Al_x_Ga_1−x_)_0.5_In_0.5_P alloys are expected to be equally dependent on ternary In_y_Al_l−y_P and In_y_Ga_l−y_P alloys [52,53] because they provide complementary information to the Raman scattering spectroscopy. The results of the (Al_0.24_Ga_0.76_)_0.5_In_0.5_P/GaAs epifilms were carefully analyzed theoretically (cf. Section 2.4) by using a three-phase ‘air–film–substrate’ model in the framework of a classical ‘Drude–Lorentz’ approach [64], which confirmed the linking of the observed phonon spectral features to the InP-, GaP-, and AlP-like modes, where the role of the GaAs substrate cannot be ignored.

### 2.4. Simulation of Reflectivity Spectra in the FIR Region

In the FIR region, the physical process involved in polar compounds can be described in terms of the interactions between photons and the crystal lattice by using a wave-vector $\vec{q}$ and frequency-dependent dielectric response function $\tilde{\varepsilon }(\omega ,\vec{q}).$ In semiconductor materials, there are two main processes that contribute to $\tilde{\varepsilon }(\omega ,\vec{q})$: (a) the lattice effect [${\tilde{\varepsilon }}_{lat}(\omega ,\vec{q})$] from the optical phonons, and (b) the free-carrier effect [${\tilde{\varepsilon }}_{fc}(\omega ,\vec{q})$] from electrons in the conduction band or holes in the valence band. In the long-wavelength limit $\vec{q}\to \;$0, the complex $\tilde{\varepsilon }\left(\omega \right)\;in\;the\;classical\;‘Drude—Lorentz’\;scheme$ holds with sufficient accuracy for the description of contributions from free carriers to the lattice phonons in alloy semiconductors. For evaluating $\tilde{\varepsilon }\left(\omega \right)$, the model can be expressed as [64]

(3) $$\tilde{\varepsilon }(\omega )={\tilde{\varepsilon }}_{lat}(\omega )+{\tilde{\varepsilon }}_{fc}(\omega )=\varepsilon_{\infty }+\sum_{j}\frac{S_{j}\omega_{j}^{2}}{\omega_{j}^{2}-\omega^{2}-i\gamma_{j}\omega }-\varepsilon_{\infty }\frac{\omega_{p}^{2}}{\omega (\omega +\frac{i}{\tau })},$$

where ${\tilde{\varepsilon }}_{lat}\left(\omega \right)(\equiv \varepsilon_{\infty }+\sum_{j}\frac{S_{j}\omega_{j}^{2}}{\omega_{j}^{2}-\omega^{2}-i\gamma_{j}\omega })$; ${\tilde{\varepsilon }}_{fc}(\omega )(\equiv \varepsilon_{\infty }\frac{-\omega_{p}^{2}}{\omega \left(\omega +i\gamma_{p}\right)})$. The term $\omega_{p}=\sqrt{\frac{4\pi \eta e^{2}}{{m_{e}}^{*}\varepsilon_{\infty }}}$ represents the plasma frequency; η stands for the free-charge carrier concentration; ${m_{e}}^{*}$ is the effective mass; $\gamma_{p}(\equiv \frac{1}{\tau }\equiv \frac{e}{m^{*}\mu })$ indicates the plasma damping constant; and μ is the mobility. The modeling of Equation (3) for the quaternary alloys requires appropriate parameters such as $S_{j}$, $\gamma_{j},$ and $\omega_{j}$ (see Table 4A), which run over the index j, with j ≤ 3 and for the substrate (see Table 4B). For further information about the history and applicability of $\tilde{\varepsilon }\left(\omega \right)$, the reader is referred to an earlier publication [64] with appropriate discussions.

To comprehend the reflectivity spectra of thin quaternary alloy (Al_0.24_Ga_0.76_)_0.5_In_0.5_P epifilms grown on the n^+^-GaAs substrate, we adopted here a three-phase (i.e., ‘air–film–substrate’) model. In this scheme, one considers the dielectric functions of the air $\varepsilon_{1}=1,$ thin film $\varepsilon_{2}={\tilde{\varepsilon }}_{tf}$, and substrate $\varepsilon_{3}={\tilde{\varepsilon }}_{s}$. Following Cadman and Sadowski [72], the reflection ${\tilde{r}}_{123}$ coefficient at near-normal incidence for an epilayer of thickness d can be obtained by using [64]

(4) $${\tilde{r}}_{123}=\frac{{\tilde{r}}_{12}+{\tilde{r}}_{23}\exp[i2\beta ]}{1+{\tilde{r}}_{12}{\tilde{r}}_{23}\exp[i2\beta ]},$$

where ${\tilde{r}}_{ij}=\frac{{\tilde{n}}_{i}-{\tilde{n}}_{j}}{{\tilde{n}}_{i}+{\tilde{n}}_{j}}$ represents the Fresnel coefficients; $\beta =2\pi d\omega \sqrt{{\tilde{\varepsilon }}_{2}}$ signifies the phase multiplier; and d is the film thickness. In terms of ${\tilde{r}}_{123}$, the power reflection $R\left(\omega \right)$ can be calculated by using [64]

(5) $$R(\omega )={\left|{\tilde{r}}_{123}\right|}^{2}.$$

**Figure 5:**
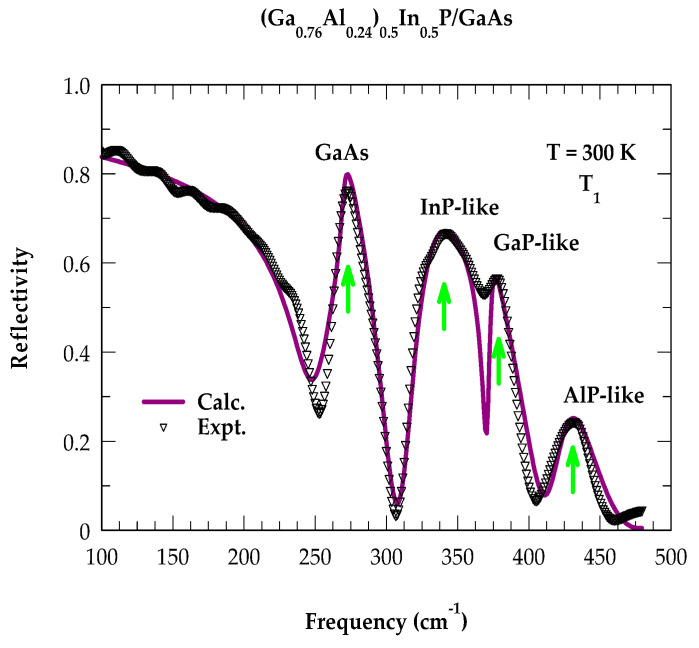
Comparison of the simulated (violet-colored solid line) IR reflectivity spectra $R\left(\omega \right)$ with the experimental (black-colored inverted triangles) data for the (Al_x_Ga_1−x_)_0.5_In_0.5_P/n^+^-GaAs epilayer (sample T_1_). The simulation was performed using a three-phase ‘air–film–substrate’ model (Ref. [64]), while experimental data were obtained by a high-resolution Brüker IFS 120 v/S FTIR spectrometer (see text).

To obtain ${\tilde{\varepsilon }}_{tf}(\omega )$ for an ultrathin MOCVD-grown (Al_0.24_Ga_0.76_)_0.5_In_0.5_P epifilm (sample #T_1_) and ${\tilde{\varepsilon }}_{s}\left(\omega \right)$ for the GaAs substrate, we used Equation (3) and incorporated appropriate values of the parameters listed in Table 4A,B. By employing Equations (4) and (5), the FIR reflectivity spectra $R\left(\omega \right)$ for sample #T_1_ are simulated in the frequency region 50 cm^−1^–600 cm^−1^ for the (Al_0.24_Ga_0.76_)_0.5_In_0.5_P/n^+^-GaAs epilayer (see Figure 5). The theoretical result of reflectivity revealing a ‘three-mode behavior’ agrees reasonably well with the experimental data, implying their role in establishing a good-quality epilayer.

### 2.5. Analysis of Optical Spectra for Quaternary Alloys

#### 2.5.1. Dielectric Function in the NIR → UV Energy Region

The accurate determination of the epilayer thickness d has been and still is a major challenge for both scientists and engineers. Reflectivity $R\left(E\right)$ spectra in the NIR → UV energy regions have frequently been used for appraising d of different nanostructured films. By using traditional methods, the accurate assessment of film thickness is not feasible due to significant reductions of interference fringes in the transparent regions. Quite recently, Ramírez et al. [73] adopted the TMM approach for effectively estimating d in various nanostructured Zn(Cd)Te/GaAs epilayers by comparing the simulated $R\left(E\right)$ spectra with the experimental data. In the TMM, one is required to have comprehensive energy-dependent dispersions of the optical constants [$\tilde{\varepsilon }(E$) and/or $\tilde{n}(E$)] for both the epilayers and substrates.

In Section 2.5.2, we succinctly outlined the salient features of the TMM for evaluating $R\left(E\right)$ and $T\left(E\right)$ in semiconductor epilayers. By considering a macroscopic MDF approach and carefully incorporating the estimated values of inter-band transition energies at critical points from the SE measurements [29,44,74], we (cf. Section 2.5.3) have simulated the optical constants for the quaternary (Al_x_Ga_1−x_)_0.5_In_0.5_P alloys and GaAs substate. The results of both epifilm and substrate were systematically incorporated for calculating the $R\left(E\right)$ and $T\left(E\right)$ spectra of a 0.9 μm thick (Al_0.24_Ga_0.76_)_0.5_In_0.5_P/GaAs epilayer (see Section 2.5.4). The analysis of the $R\left(E\right)$/$T\left(E\right)$ data provided the energy bandgap $E_{g}^{PL}$ for the quaternary alloy in excellent agreement with the room-temperature PL data, signifying the quality of the MOCVD-grown epifilm.

#### 2.5.2. Transfer Matrix Method

An ultrathin film of any material is regarded as a plane-parallel layer to an infinite extent. The most satisfactory foundation of calculating optical properties in thin-film optics is derived from an electromagnetic (EM) theory. In this formalism, all energy relations are expressed in terms of the steady-state amplitudes of both the electric and magnetic field vectors at the successive interfaces of multilayers [29].

An alternative way of simulating the optical response in a multilayer system is to consider the TMM. In this formalism, we succinctly outlined the methodology adopted for calculating $R\left(E\right)$/$T\left(E\right)$ spectra in multilayer epifilms. For an epilayer of thickness d, the characteristic matrix M (or Fresnel matrix) in the TMM can be expressed as [73]

(6) $$M\equiv \left(\begin{array}{cc} \cos\;\delta & \frac{i\;sin\;\delta }{\eta }\\ i\;sin\;\delta & cos\;\delta \end{array}\right)$$

where

(7a) $$\delta \;\equiv \;\left(\frac{2\pi \tilde{n}d}{\lambda }cos\;\theta \right),$$

(7b) $$\eta \equiv \left\{\begin{array}{c} \;\;\;\left(\;\tilde{n}\;cos\;\theta \right)\;is\;for\;s-polarization\\ \left(\frac{\tilde{n}}{cos\;\theta }\right)\;is\;for\;p-polarization \end{array}\right.$$

and θ is the angle of incidence.

The second-order matrix in Equation (6) is commonly expressed as

(8) $$M\equiv \left(\begin{array}{cc} m_{11} & m_{12}\\ m_{21} & m_{22} \end{array}\right).$$

In this approach and using Equation (8), the amplitude of reflectance ($\tilde{r}$) and transmittance ($\tilde{t}$) coefficients are derived as a function of the matrix components [73]:

(9) $$\tilde{r}\equiv \frac{\left(\eta_{0}m_{11}+\eta_{0}{\eta_{s}m}_{12}\right)-\left(m_{21}+{\eta_{s}m}_{22}\right)}{\left(\eta_{0}m_{11}+\eta_{0}{\eta_{s}m}_{12}\right)+\left(m_{21}+{\eta_{s}m}_{22}\right)},$$

and

(10) $$\tilde{t}\equiv \frac{2\eta_{0}}{\left(\eta_{0}m_{11}+\eta_{0}{\eta_{s}m}_{12}\right)+\left(m_{21}+{\eta_{s}m}_{22}\right)}.$$

where $\eta_{0}$ and $\eta_{s}$ signify the coefficients that are identical to those reported by Hecht [75] and characterize the optical properties of the epifilm and substrate, respectively. Multiplying ($\tilde{r}$) and ($\tilde{t}$) by their complex conjugates can lead to the reflectance:

(11) $$R(E)\equiv \left|{\tilde{r}}^{2}\right|,$$

and transmission:

(12) $$T(E)\equiv \left|{\tilde{t}}^{2}\right|.$$

#### 2.5.3. Optical Constants in NIR → UV Region for (Al_x_Ga_1−x_)_0.5_In_0.5_P

Several macroscopic methodologies exist in the literature for comprehending the optical properties of different semiconductor materials [29]. Appropriate analytical expressions have been developed in various theoretical schemes by carefully incorporating the inter-band transition energies at the critical points in the BZ. Frequently adopted methods for simulating the optical constants in binary/ternary/quaternary semiconductor alloys include (i) DHOs, (ii) SCP, and (iii) MDFs. These methodologies have enabled researchers to obtain $\tilde{\varepsilon }(E$) and/or $\tilde{n}(E$) in the NIR → UV energy regions for assessing optical traits in different semiconductor materials [29]. In the framework of an MDF approach by appropriately including contributions of the high-energy inter-band transitions at critical points [29,44,74], we simulated the energy-dependent dispersions of $\tilde{\varepsilon }(E)$ and $\tilde{n}(E$) for both the quaternary (Al_x_Ga_1−x_)_0.5_In_0.5_P alloys and the GaAs substrate.

**Figure 6:**
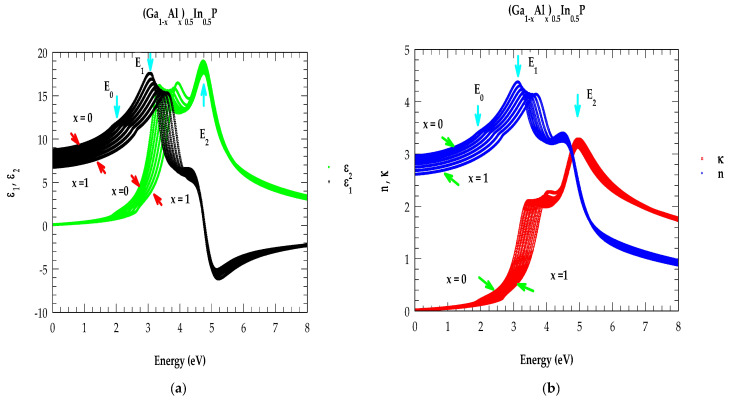
(**a**) Simulation of E-dependent $\varepsilon_{1}(E)$, $\varepsilon_{2}(E)$ for the (Al_x_Ga_1−x_)_0.5_In_0.5_P alloys with an increment of composition x. (**b**) Simulation of E-dependent n(E), κ(E) for the (Al_x_Ga_1−x_)_0.5_In_0.5_P alloys with an increment of composition x.

In Figure 6a,b, the calculated results of $\tilde{\varepsilon }(E)$ and $\tilde{n}(E$) are displayed for the quaternary (Al_x_Ga_1−x_)_0.5_In_0.5_P alloys in the energy range of 0–8 eV by using an increment of the alloy composition x (≡0.2). Here, we used different color points for indicating the simulated results of $\varepsilon_{1}$(E) (black color points), $\varepsilon_{2}$(E) (green color points),$\;n\left(E\right)$ (blue color points), and κ(E) (red color points). Clearly, the calculated results validated the experimental SE measurements [29,44,74] by achieving excellent agreements. Moreover, the perusal of Figure 6a,b shows that the spectra of both $\tilde{\varepsilon }(E)$ and $\tilde{n}(E$) shift towards the high-energy side as the alloy composition x increases, in accordance with the change in inter-band transition energies ($E_{0},E_{1},E_{2},\;etc.$) at high critical points in the BZ. We also noticed (see Figure 6b) that the strongest peaks in κ(E) are related to the $E_{2}$ transitions.

#### 2.5.4. Thickness-Dependent Reflectivity and Transmission Spectra

**Figure 7:**
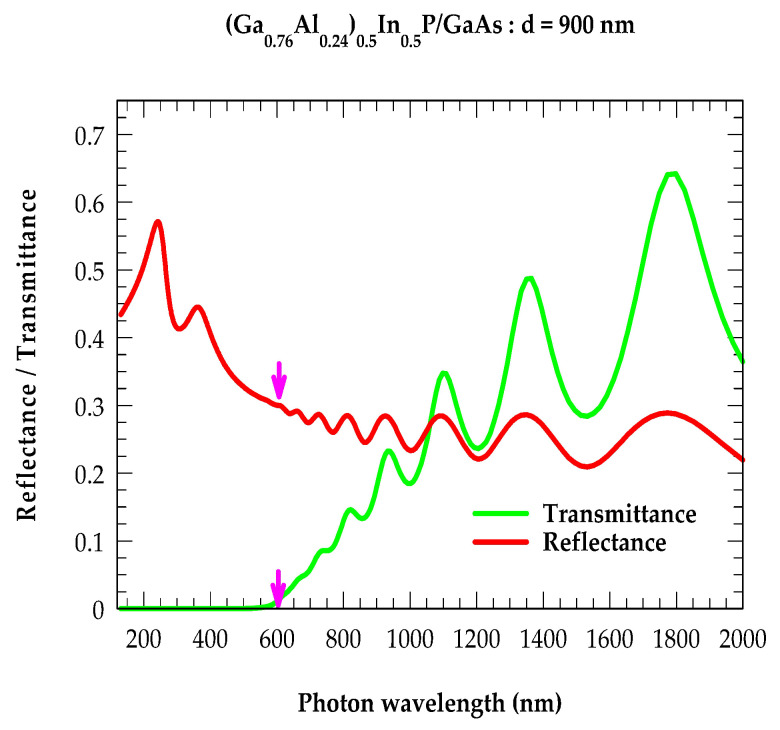
Transfer-matrix-based simulated spectra of reflectance R(E) (red-colored line) and transmission T(E) (green-colored line) for the 0.9 μm thick (Al_0.23_Ga0.74)_0.5_In_0.5_P epifilm grown on the GaAs substrate. The magenta-colored vertical arrows drawn near 605 nm predicted the bandgap of ~2.05 eV, in excellent agreement with our PL and existing data from the literature [71].

Recently, Ramírez et al. [73] employed the TMM approach and successfully tested its use for accurately estimating the film thicknesses in nanometer-sized Zn(Cd)Te/GaAs epilayers. The authors [73] simulated reflectance $R\left(E\right)$ spectra by methodically incorporating the optical constants [n(E),$\;\kappa \left(E\right)$] of both the epilayers and substrate. A comparison of $R\left(E\right)$ with the experimental data offered a simple way of assessing the accuracy of a given film thickness d. Here, we explored the use of the TMM for simulating both $R\left(E\right)$ and $T\left(E\right)$ for achieving the accurate energy bandgap values of the nanometer-sized (Al_x_Ga_1−x_)_0.5_In_0.5_P/GaAs epifilms. The simulated results for the optical constants (cf. Section 2.5.3) of the epilayer, substrate, and film thickness d were carefully integrated in the methodology outlined in Section 2.5.2 for acquiring the reflectivity R(E) and transmission T(E) spectra at near-normal incidence θ = 0. In Figure 7, the calculated results of R(E) and T(E) spectra for the (Al_x_Ga_1−x_)_0.5_In_0.5_P/GaAs epilayer are displayed for the film of thickness d [≡900 nm (or 0.9 μm)] by using different-colored lines (red: reflectance; green: transmittance). Vertical magenta-colored arrows drawn near 605 nm ($E_{g}^{PL}$≡2.05 eV) in Figure 7 clearly show the bandgap of the (Al_0.24_Ga_0.76_)_0.5_In_0.5_P/GaAs epilayer, in excellent agreement with our TD-PL and existing data from the literature [71].

## 3. Material Growth and Characterization Methods

### 3.1. MOCVD Growth of (Al_x_Ga_1−x_)_0.5_In_0.5_P/n^+^-GaAs

The needs of designing different electronic devices based on (Al_x_Ga_1−x_)_y_In_1−y_P alloys require epitaxially grown films, MQWs, and SLs on appropriate lattice-matched substrates. The flexibility of selecting different compositions x, y and film thicknesses d comes at the expense of difficult growth processes coupled with many requisites of using tedious calibration methods. A low-pressure MOCVD method in the vertical growth configuration is used here for preparing a set of four T_i_ (i = 1 to 4) (Al_0.24_Ga_0.76_)_0.5_In_0.5_P/n^+^-GaAs samples. High-purity trimethyl indium (TM In), trimethyl gallium (TM Ga), and trimethyl aluminum (TM Al) were employed as precursors to supply In, Ga, and Al, respectively, while using PH_3_ to provide P. High-purity H_2_ was exploited as a carrier gas. Following the methodology described elsewhere [57], the (Al_0.24_Ga_0.76_)_0.5_In_0.5_P epifilms of thickness d (≡0.85 μm–1.1 μm) were prepared under different conditions by aligning an n^+^-GaAs substrate at 15^o^ with respect to the nearest <110> direction. Under appropriate conditions, the samples were grown at 690 °C by maintaining suitable pressures τ, H_2_-flow rates, and n-type dopants (see Table 1). An Olympus BX51 Nomarski interference microscope and EDX spectroscopy were employed to examine the structural and chemical distribution of the atoms on epifilms [57]. Methodical analyses of PL, RSS, and FTIR measurements (cf. Section 2.1, Section 2.2, Section 2.3, Section 2.4 and Section 2.5) were accomplished for assessing the impact of x, y and d on optical and vibrational characteristics to evaluate the quality of the MOCVD-grown samples.

### 3.2. Photoluminescence

Photoluminescence is a simple and non-destructive method for evaluating the electronic energy bandgaps ($E_{g}^{PL}$) of binary, ternary, and/or quaternary alloys. Systematic measurements of temperature-dependent PL studies were conducted on MOCVD-prepared (Al_0.24_Ga_0.76_)_0.5_In_0.5_P/n^+^-GaAs epilayers. A 532 nm Nd:YAG laser (cf. Section 2.1) was used as an excitation source by calibrating the radiation lines of a Xe lamp and employing a HORIBA Jobin Yvon T64000 micro-Raman system equipped with a charge-coupled device and an InSb detector.

### 3.3. Raman Scattering

Raman scattering spectroscopy is frequently used for comprehending the lattice dynamics of semiconducting alloys. We performed RSS measurements in the backscattering geometry on (Al_0.24_Ga_0.76_)_0.5_In_0.5_P/n^+^-GaAs epilayers by using a Renishaw Raman Microscope (model-100) with a 633 nm line from a He–Ne laser. A holographic notch filter was employed to block unwanted reflections. The phonon traits exhibited strong dependence on their structural (e.g., x, y and d) features. One must note that Raman selection rules in zincblende (zb) crystals with a (100) surface forbid the observation of the $\omega_{TO}$ mode and allow only the $\omega_{LO}$ phonon [54,55]. Misalignment or disorder in samples can, however, relax these selection rules. The intensity ratio between the allowed $\omega_{LO}$ and forbidden $\omega_{TO}$ modes is used for carefully characterizing the quality of the samples. Composition-dependent optical phonons for (Al_0.24_Ga_0.76_)_0.5_In_0.5_P/n^+^-GaAs epilayers were obtained in the frequency range of ~40 cm^−1^ to 800 cm^−1^. Several scans were performed on each sample at different locations with a run of 16 accumulations for reducing the impacts of noise. Despite setting an exposure time of ~10 s to minimize the thermal effects on each sample, it remained inevitable for local heating to cause the broadening in the observed phonon bands. The complex mode behavior (see Section 2.2) in the (Al_0.24_Ga_0.76_)_0.5_In_0.5_P alloys was found to be dependent on the Raman scattering spectroscopy data of the ternary In_y_Al_l−y_P and In_y_Ga_l−y_P alloys.

### 3.4. Infrared Spectroscopy

Room-temperature (RT) FTIR reflectivity measurements were performed on each MOCVD (Al_0.24_Ga_0.76_)_0.5_In_0.5_P/n^+^GaAs grown epifilm (cf. Section 3.1) by using a high-resolution Brüker IFS 120 v/S FTIR spectrometer with a 2 cm^−1^ resolution and 100 coadditions. In these studies, we employed a Globar-source, high-efficiency Mylar 6 beam-splitter and a Mercury Cadmium Telluride (MCT) detector. Unpolarized IR reflectivity experiments are also carried out using a deuterated triglycine sulfate (DTGS 201) detector. FTIR spectroscopy is an alternative technique to RSS for determining the lattice phonons in semiconductor materials. In (Al_0.24_Ga_0.76_)_0.5_In_0.5_P samples, the method offered a ‘three-mode behavior’ (see: Section 2.3), where the effects of the GaAs substrate cannot be ignored.

## 4. Concluding Remarks

The knowledge of the optical, structural, and phonon properties of a single, ultrathin (Al_x_Ga_1−x_)_0.5_In_0.5_P/GaAs epilayer and/or multilayers are considered of paramount importance for designing different LDH-based optoelectronic, photonic, and multi-junctional photovoltaic devices. By using TD-PL, RSS, and high-resolution FTIR spectroscopy, we reported the results of our systematic studies of electrical, structural, and phonon characteristics of MOCVD-grown highly disordered quaternary AlGaInP epilayers prepared on lattice-matched n^+^-GaAs substrates. Macroscopic models were meticulously employed for analyzing the experimental data for assessing their appropriate energy-dependent [E(≡ħ*ω*)] optical responses. In the FIR region, we examined the reflectivity spectra of the (Al_0.24_Ga_0.76_)_0.5_In_0.5_P/n^+^-GaAs epilayer at near-normal incidence (θ = 0) by using a classical three-phase ‘Drude–Lorentz’ model (‘air–film–substrate’) within an effective medium approximation. Apposite InP-, GaP-, and AlP-like optical phonon energies are shown, providing dominant contributions for analyzing the experimental FTIR reflectivity spectra. Except for the observed GaP-like mode feature, our simulations agreed reasonably well with the experimental results. Moreover, the phonon features observed in our RSS are complimentary to the FTIR results. Slight scattering in the phonon frequency values of AlP- and GaP-like modes noticed in our and other existing studies is not surprising, as these vibrations are likely subsumed by the strong influence of InP-like phonons in the RSS measurements. In the NIR → UV energy regions and adopting an MDF formalism, we accurately simulated the optical constants (i.e., n(E), κ(E)) of both the Al_0.24_Ga_0.76_)_0.5_In_0.5_P film and the GaAs substrate. These results are drawn from the spectroscopic ellipsometry features of the inter-band transitions at high-energy critical points in the BZ. By meticulously incorporating these optical constants in the TMM, we systematically evaluated the reflectivity R(E) and transmission T(E) spectra of the ultrathin Al_0.24_Ga_0.76_)_0.5_In_0.5_P/n^+^-GaAs epilayer for validating the TD-PL results of the bandgap $E_{g}^{PL}$ energy of an ultrathin film. We strongly believe that the optical, structural, and phonon characterization results reported here for the AlInGaP quaternary alloys can be extended to other low-dimensional heterostructure materials of technological interest.

## Figures and Tables

**Table 1:** Thickness-dependent (d, μm), room-temperature (RT) transport, and electrical characteristics of four T_i_ (i = 1 to 4) MOCVD-grown (Ga_1−x_Al_x_)_0.5_In_0.5_P/n^+^ GaAs samples with x = 0.24. The charge carrier concentration η (10^18^ cm^−3^), mobility μ (cm^2^/Vs), effective electron mass m*/m_e_, pressure, flow rate of H_2_, and dopants are reported. These parameters played important roles for studying the optical and vibrational properties (see text).

Samples	d (μm)	η	μ	m*/m_e_	Pressure Torr τ	H_2_ Flow Rate (slm)	Dopant
T_1_	0.98	0.91	596.17	0.13	35	85	Si
T_2_	1.1	1.03	664.74	0.13	55	85	Si
T_3_	0.85	2.59	321.38	0.13	55	120	Te
T_4_	1.08	~0.01	672.29	0.13	70	85	

**Table 2:** Our Raman scattering spectroscopy measurements in four T_i_ (i = 1 to 4) MOCVD-grown (Ga_1−x_Al_x_)_0.5_In_0.5_P/n^+^GaAs samples with x = 0.24 (cf. Section 3.1) have revealed the optical phonon frequencies.

Samples	d (μm)	GaA-like	InP-like		GaP-like		AlP-like	
		$\omega_{TO}$	$\omega_{TO}$	$\omega_{LO}$	$\omega_{TO}$	$\omega_{LO}$	$\omega_{TO}$	$\omega_{LO}$
T_1_	0.98	268.5	330.3	365.0	-	375.2	-	453.0
T_2_	1.1	269.1	329.9	365.1	-	385.0	-	465.0
T_3_	0.85	269.0	329.8	365.3	-	371.3	-	441.0
T_4_	1.08	268.7	330.2	365.3	-	386.1	-	455.0

**Table 3:** The results of FTIR spectroscopy measurements revealed optical phonons in four T_i_ (i = 1 to 4) MOCVD-grown (Ga_1−x_Al_x_)_0.5_In_0.5_P/n^+^ GaAs samples (cf. Section 3.1) with x = 0.24.

Samples	d (μm)	GaAs-like $\omega_{TO}$	InP-like $\omega_{TO}$	GaP-like $\omega_{TO}$	AlP-like $\omega_{TO}$
T_1_	0.98	268.7	335.3	372.3	422.3
T_2_	1.1	269.2	335.2	375.2	422.4
T_3_	0.85	269.0	335.1	365.3	422.3
T_4_	1.08	268.4	324.2	390.1	418.1

**Table 4:** The optical parameters are obtained by meticulously fitting the room-temperature experimental FIR reflectivity spectra for the MOCVD-grown (Ga_1−x_Al_x_)_0.5_In_0.5_P/GaAs sample T_1_ of d = 0.98 μm with x = 0.24. (**A**) The necessary parameters (ε_∞j_, Sj, ω_Toj,_, and γ_j’s_) of InP-like, GaP-like, and AlP-like modes are carefully derived by using a ‘Drude–Lorentz’-type model in the framework of a ‘three-phase multilayer’ optics methodology. (**B**) The parameters used in the calculation of reflectivity spectra for the GaAs substrate (see text).

(**A**)		
**(Ga_1−x_Al_x_)_0.5_In_0.5_P/n^+^GaAs Parameters** **x = 0.24** 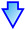		**Sample T_1_** 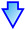
	ε_∞_	8.81
InP-like mode	S_1_	1.76
	$\omega_{TO1}$ (cm^−1^)	335.57
	γ_1_ (cm^−1^)	16.28
GaP-like mode	S_2_	0.11
	$\omega_{TO2}$ (cm^−1^)	372.34
	γ_2_ (cm^−1^)	4.30
AlP-like mode	S_3_	0.31
	$\omega_{TO3}$ (cm^−1^)	422.30
	γ_3_ (cm^−1^)	23.22
(**B**)		
**n^+^ GaAs** **Substrate** **300 K**		
ε_∞_	10.89	
S	4.53	
$\omega_{TO}$ (cm^−1^)	268.51	
γ (cm^−1^)	5.05	
Carrier concentration (10^18^ cm^−3^)	1.14	
Mobility (cm^2^/Vs)	1383.12	
Effective mass (m*/m_e_)	0.063	

## Data Availability

The data that support the findings of this study are available from the corresponding author upon reasonable request.

## References

[1] Xie L., Li S., Bi J., Xue L., Wang Y., Lai Y., Liao Y., Dong X., Yang M., Wang B. (2023). Effect of window layer with different growth modes on the photoelectric properties of AlGaInP LED. AIP Adv..

[2] Brückner S., Maaßdorf A., Weyers M. (2022). In situ control of indium incorporation in (AlGa)_1−x_In_x_P layers. J. Cryst. Growth.

[3] Zhang S., Li X., Li G., Lu H., Wang X. (2022). Design of Quadruple-Layer Antireflection Coating for AlGaInP/AlGaAs/GaAs Three-Junction Solar Cell. Energy Technol..

[4] Li G., Lu H., Li X., Zhang W. (2022). Improving the Performance of Direct Bonded Five-Junction Solar Cells by Optimization of AlInP Window Layer. Photonics.

[5] Li Y.-Y., Lin F.-Z., Chi K.-L., Weng S.-Y., Lee G.-Y., Kuo H.-C., Lin C.-C. (2022). Analysis of Size-Dependent Quantum Efficiency in AlGaInP Micro–Light-Emitting Diodes with Consideration for Current Leakage. IEEE Photonics J..

[6] Sun Y., Fan S., Faucher J., Hool R.D., Li B.D., Dhingra P., Lee M.L. (2021). 2.0–2.2 eV AlGaInP solar cells grown by molecular beam epitaxy. Sol. Energy Mater. Sol. Cells.

[7] Ochoa-Martínez E., Barrutia L., Ochoa M., Barrigón E., García I., Rey-Stolle I., Algora C., Basa P., Kronome G., Gabás M. (2018). Refractive indexes and extinction coefficients of n- and p-type doped GalnP, AllnP and AlGalnP for multijunction solar cells. Sol. Energy Mater. Sol. Cells.

[8] Horng R.-H., Chien H.-Y., Chen K.-Y., Tseng W.-Y., Tsai Y.-T., Tarntair F.-G. (2018). Development and Fabrication of AlGaInP-Based Flip-Chip Micro-LEDs. J. Elect. Devices Soc..

[9] Ledentsov N., Shchukin V., Shernyakov Y., Kulagina M., Payusov A., Gordeev N., Maximov M., Zhukov A., Denneulin T., Cherkashin N. (2018). Room-temperature yellow-orange (In,Ga,Al)P–GaP laser diodes grown on (n11) GaAs substrates. Opt. Exp..

[10] Schulte K.L., Simon J., Ptak A.J. (2018). Multijunction Ga_0.5_In_0.5_P/GaAs solar cells grown by dynamic hydride vapor phase epitaxy. Prog. Photovolt. Res Appl..

[11] Streubel K. (2017). Light-emitting diodes (LEDs). Handbook of Optoelectronics.

[12] Ledentsov N.N., Shchukin V.A., Shernyakov Y.M., Kulagina M.M., Payusov A.S., Gordeev Y.N., Maximov M.V., Cherkashin N.A. (2017). (In,Ga,Al)P–GaP laser diodes grown on high index GaAs surfaces emitting in the green, yellow and bright red spectral range. Semicond. Sci. Technol..

[13] Cheong J.S., Baharuddin A.N.A.P., Ng J.S., Krysa A.B., David J.P.R. (2017). Absorption coefficients in AlGaInP lattice matched to GaAs. Sol. Energy Mater. Sol. Cells.

[14] Berg A., Yazdi S., Nowzari A., Storm K., Jain V., Vainorius N., Samuelson L., Wagner J.B., Bergstrom M.T. (2016). Radial Nanowire Light-Emitting Diodes in the (Al_x_Ga_1−x)_)_y_In_1−y_P Material System. Nano Lett..

[15] Vaisman M., Mukherjee K., Masuda T., Yaung K.N., Fitzgerald E.A., Lee M.L. (2016). Direct-Gap 2.1–2.2 eV AlInP solar cells on GaInAs/GaAs metamorphic buffers. IEEE J. Photovolt..

[16] Pohl J., Bugge F., Blume G., Knigge A., Knigge S., Erbert G., Weyers M. (2015). Combined Mg/Zn p-type doping for AlGaInP laser diodes. J. Cryst. Growth.

[17] Masuda T., Tomasulo S., Lang J.R., Lee M.L. (2015). Comparison of single junction AlGaInP and GaInP solar cells grown by molecular beam epitaxy. J. Appl. Phys..

[18] Masuda T., Tomasulo S., Lang J.R., Lee M.L. Effect of substrate effcut angle on AlGaInP and GaInP solar cells grown by molecular beam epitaxy. Proceedings of the 2014 IEEE 40th Photovoltaic Specialist Conference (PVSC).

[19] Shin J.W., Jeong H.Y., Yoo S.J., Lee S.-H., Han J.H., Lee J.Y., Ahn J.S., Park C.Y., Park K.W., Lee Y.-T. (2014). Atomic variations in digital alloy InGaP/InGaAlP multiple quantum wells due to thermal treatment. Jpn. J. Appl. Phys..

[20] Rossbach R., Schulz W.M., Reischle M., Beirne G.J., Jetter M., Michler P. (2007). Red to green photoluminescence of InP-quantum dots in Al_x_Ga_1−x_InP. J. Cryst. Growth.

[21] Liu C.Y., Yuan S., Dong J.R., Chua S.J. (2004). Temperature dependence of photoluminescence intensity from AlGaInP/GaInP multi-quantum well laser structures. J. Cryst. Growth.

[22] Chen L., Fan G., Meng Y. (2004). Study of the long-wavelength optic phonons in AlGaInP and AlGaInAs. Microelectron. J..

[23] Lu T.C., Shieh H.M., Wang S.C. (2002). Real index-guided InGaAlP red lasers with buried tunnel junctions. Appl. Phys. Lett..

[24] Vanderwater D.A., Tan I.-H., Hofler G.E., Defevere D.C., Kish F.A. (1997). High-Brightness AlGaInP Light Emitting Diodes. Proc. IEEE.

[25] Streubel K., Linder N., Wirth R., Jaeger A. (2002). High Brightness AlGaInP Light-Emitting Diodes. IEEE J. Sel. Top. Quantum Electronics.

[26] Kish F.A., Steranka F.M., DeFevere D.C., Vanderwater D.A., Park K.G., Kuo C.P., Osentowski T.D., Peanasky M.J., Yu J.G., Fletcher R.M. (1994). Very high-efficiency semiconductor wafer-bonded transparent-substrate (Al_x_Ga_1−x_)_0.5_ In_0.5_P/GaP light-emitting diodes. Appl. Phys. Lett..

[27] Kish F.A., Vanderwater D.A., DeFevere D.C., Steigerwald D.A., Hofler G.E., Park K.G., Steranka F.M. (1996). Highly reliable and efficient semiconductor wafer-bonded AlGaInP/GaP light-emitting diodes. Electron. Lett..

[28] Ryou J.H., Dupuis R.D., Walter G., Holonyak N., Mathes D.T., Hull R., Reddy C.V., Narayanamurti V. (2002). Properties of InP self-assembled quantum dots embedded in In_0.49_(Al_x_Ga_1−x_)_0.51_P for visible light emitting laser applications grown by metalorganic chemical vapor deposition. J. Appl. Phys..

[29] Adachi S. (2009). Properties of Semiconductor Alloys: Group-IV, III–V and II–VI Semiconductors.

[30] Donati G.P., Kaspi R., Malloy K.J. (2003). Interpolating semiconductor alloy parameters: Application to quaternary III–V band gaps. J. App. Phys..

[31] Cripps S.A., Hosea T.J.C., Krier A., Smirnov V., Batty P.J., Zhuang Q.D., Lin H.H., Liu P.W., Tsai G. (2007). Mid-infrared photoreflectance study of InAs-rich InAsSb and GaInAsPSb, indicating negligible bowing for the spin-orbit splitting energy. Appl. Phys. Lett..

[32] Asahi H., Emura S., Gonda S., Kawamura Y., Tanaka H. (1989). Raman scattering in InGaAlP layers grown by molecular-beam epitaxy. J. Appl. Phys..

[33] Tsai G., Wang D.L., Wu C.E., Wu C.J., Lin Y.T., Lin H.H. (2007). InAsPSb quaternary alloy grown by gas source molecular beam epitaxy. J. Cryst. Growth.

[34] Kondo M., Okada N., Domen K., Sugiura K., Anayama C., Tanahashi T. (1994). Origin of nonradiative recombination centers in AlGaInP grown by metalorganic vapor phase epitaxy. J. Electron. Mater..

[35] Perl E.E., Simon J., Geisz J.F., Olavarria W., Young M., Duda A., Friedman D.J., Steiner M.A. (2016). Development of high-bandgap AlGaInP solar cells grown by organometallic vapor-phase epitaxy. IEEE J. Photovolt..

[36] Perl E.E., Simon J., Geisz J.F., Olavarria W., Young M., Duda A., Dippo P., Friedman D.J., Steiner M.A. Development of a 2.0 eV AlGaInP Solar Cell Grown by OMVPE. Proceedings of the 42nd IEEE Photovoltaics Specialists Conference (PVSC).

[37] Kondow M., Minagawa S. (1988). Study on photoluminescence and Raman scattering of GaInP and AlInP grown by organometallic vapor-phase epitaxy. J. Appl. Phys..

[38] Hofmann T., Schubert M., Gottschalch V. (2004). Far-infrared dielectric function and phonon modes of spontaneously ordered (Al_x_Ga_(1−x)_)_0.52_In_0.48_P. Thin Solid Film..

[39] Hofmann T., Leibiger G., Gottschalch V., Pietzonka I., Schubert M. (2001). Infrared dielectric function and phonon modes of highly disordered (Al_x_Ga_(1−x)_)_0.52_In_0.48_P. Phys. Rev. B.

[40] Mukherjee K., Deotare P.B., Fitzgerald E.A. (2015). Improved photoluminescence characteristics of order-disorder AlGaInP quantum wells at room and elevated temperatures. Appl. Phys. Lett..

[41] Prins A.D., Sly J.L., Meney A.T., Dunstan D.J., O’reilly E.P., Adams A.R., Valster A. (1995). High pressure determination of AlGaInP band structure. J. Phys. Chem. Solids.

[42] Borrott R., Merlin R., Chin A., Bhattacharya P.K. (1988). Raman scattering by optical phonons in In_1−y−z_Al_y_Ga_z_As lattice matched to InP. Appl. Phys. Lett..

[43] Huang L.Y., Chen C.H., Chen Y.F., Yeh W.C., Huang Y.S. (2002). Degree of ordering in Al_0.5_In_0.5_P by Raman scattering. Phys. Rev. B.

[44] Lee H., Klein M.V., Aspnes D.E., Kuo C.P., Peanasky M., Craford M.G. (1993). Optical study of (Al_x_Ga_1−x_)_0.5_In_0.5_P/GaAs semiconductor alloys by spectroscopic ellipsometry. J. Appl. Phys..

[45] Grillot P.N., Stockman S.A., Huang J.W., Bracht H., Chang Y.L. (2002). Acceptor diffusion and segregation in (Al_x_Ga_1−x_)_0.51_In_0.49_P heterostructures. J. Appl. Phys..

[46] Peng H., Ailihumaer T., Liu Y., Raghothamachar B., Dudley M. (2020). Characterization of defects and strain in the (Al_x_Ga_(1−x)_)_0.5_In_0.5_P/GaAs system by synchrotron X-ray topography. J. Cryst. Growth.

[47] Ho C.-H., Li J.-H., Lin Y.-S. (2007). Optical characterization of a GaAs/(Al_x_Ga_(1−x)_)_0.5_In_0.5_P/GaAs heterostructure cavity by piezo reflectance spectroscopy. Opt. Exp..

[48] Lin C., Kelley D.F., Rico M., Kelley A.M. (2014). The “Surface Optical” Phonon in CdSe Nanocrystals. ACS Nano.

[49] Alsina F., Webb J.D., Mascarenhas A., Geisz J.F., Olson J.M., Duda A. (1999). Far-infrared transmission studies in disordered and ordered Ga_0.52_In_0.48_P. Phys. Rev. B.

[50] Pavic I., Šoda J., Gašparic V., Ivanda M. (2021). Raman and Photoluminescence Spectroscopy with a Variable Spectral Resolution. Sensors.

[51] Kondow M., Kakibayashi H., Minagawa S., Inoue Y., Nishino T., Hamakawa Y. (1988). Influence of growth temperature on crystalline structure in Ga_0.5_In_0.5_P grown by organometallic vapor phase epitaxy. Appl. Phys. Lett..

[52] Lucovsky G., Brodsky M.H., Chen M.F., Chicotka R.J., Ward A.T. (1971). Long-Wavelength Optical Phonons in Ga_1−x_In_x_P. Phys. Rev. B.

[53] Beserman R., Hirlimann C., Balkanski M., Chevallier C. (1976). Raman detection of phonon-phonon coupling in Ga_x_In_1−x_P. Solid State Commun..

[54] Jusserand B., Slempkes S. (1984). Evidence by Raman scattering on In_1−x_Ga_x_As_y_P_1−y_ of the two-mode behavior of In_1−x_Ga_x_P. Solid State Commun..

[55] Li G.H., Liu Z.X., Han H.X., Wang Z.P., Dong J.R., Wang Z.G. (1996). Raman Scattering and Photoluminescence of Spontaneously Ordered Ga_0.5_In_0.5_P Alloy. MRS Online Proc. Libr..

[56] Hofmann T., Gottschalch V., Schubert M. (2007). Dielectric anisotropy and phonon modes of ordered indirect-gap Al_0.52_In_0.48_P studied by far-infrared ellipsometry. Appl. Phys. Lett..

[57] Feng Z.C., Lin H.C., Zhao J., Yang T.R., Ferguson I. (2006). Surface and optical properties of AlGaInP films grown on GaAs by metalorganic chemical vapor deposition. Thin Solid Film..

[58] Altuieri-Weimar P., Jaeger A., Lutz T., Stauss P., Streubel K., Thonke K., Sauer R. (2008). Influence of doping on the reliability of AlGaInP LEDs. J. Mater. Sci. Mater. Electron..

[59] Jou M.-J., Lin J.-F., Chang C.-M., Lin C.-H., Wu M.-C., Lee B.-J. (1993). Metalorganic Vapor Phase Epitaxy Growth and Characterization of (Al_x_Ga_1−x_)_0.5_In_0.5_P/Ga_0.5_In_0.5_P (x = 0.4, 0.7 and 1.0) Quantum Wells on 15°-Off-(100) GaAs Substrates at High Growth Rate. Jpn. J. Appl. Phys..

[60] Borcherds P.H., Alfrey G.F., Woods A.D.B., Saunderson D.H. (1975). Phonon dispersion curves in indium phosphide. J. Phys. C Solid State Phys..

[61] Borcherds P.H., Hall R.L., Kunc K., Alfrey G.F. (1979). The lattice dynamics of gallium phosphide. J. Phys. C Solid State Phys..

[62] Orlova N.S. (1981). Variation of phonon dispersion curves with temperature in indium arsenide measured by X-ray thermal diffuse scattering. Phys. Status Solidi B.

[63] Benyahia N., Zaoui A., Madouri D., Ferhat M. (2017). Dynamic properties of III–V polytypes from density-functional theory. J. Appl. Phys..

[64] Talwar D.N., Yang T.R., Feng Z.C., Becla P. (2011). Infrared reflectance and transmission spectra in II–VI alloys and superlattices. Phys. Rev. B.

[65] Mintairov A.M., Merz J.L., Vlasov A.S. (2003). Effects of bond relaxation on the martensitic transition and optical phonons in spontaneously ordered GaInP2. Phys. Rev. B.

[66] Alsina F., Cheong H.M., Webb J.D., Mascarenhas A., Geisz J.F., Olson J.M. (1997). Far-infrared reflection studies in ordered GaInP_2_. Phys. Rev. B.

[67] Alsina F., Mestres N., Nakhli A., Pascual J. (1999). CuPt Ordering Fingerprints of Optical Phonons in Ternary III–V Compound Semiconductors. Phys. Status Solidi B.

[68] Lee H.J., Gamel M.M.A., Ker P.J., Jamaludin Z., Wong Y.H., David J.P.R. (2022). Absorption Coefficient of Bulk III-V Semiconductor Materials: A Review on Methods, Properties and Future Prospects. J. Electron. Mater..

[69] Chang I.F., Mitra S.S. (1971). Long wavelength optical phonons in mixed crystals. Adv. Phys..

[70] Varshni Y.P. (1967). Temperature dependence of the energy gap in semiconductors. Physica.

[71] Vurgaftman I., Meyer J.R., Ram-Mohan R. (2001). Band parameters for III–V compound semiconductors and their alloys. J. Appl. Phys..

[72] Cadman T.W., Sadowski D. (1978). Generalized equations for the calculation of absorptance, reflectance, and transmittance of a number of parallel surfaces. Appl. Opt..

[73] González-Ramírez J.E., Fuentes J., Hernandez L.C., Hernandez L. (2009). Evaluation of the Thickness in Nanolayers Using the Transfer Matrix Method for Modeling the Spectral Reflectivity. Res. Lett. Phys..

[74] Kato H., Adachi S., Nakanishi H., Ohtsuka K. (1994). Optical Properties of (Al_x_Ga_1−x_)_0.5_In_0.5_P Quaternary Alloys. Jpn. J. Appl. Phys..

[75] Hecht E. (1998). Optics.

